# RILP inhibits tumor progression in osteosarcoma via Grb10-mediated inhibition of the PI3K/AKT/mTOR pathway

**DOI:** 10.1186/s10020-023-00722-6

**Published:** 2023-10-03

**Authors:** Zhun Wei, Kezhou Xia, Di Zheng, Changtian Gong, Weichun Guo

**Affiliations:** Department of Orthopedics, Renmin Hospital of Wuhan University, 238 Jiefang Road, Wuhan, 430060 Hubei China

**Keywords:** RILP, Osteosarcoma, Autophagy, Epithelial–mesenchymal transition

## Abstract

**Background:**

Rab-interacting lysosomal protein (RILP) contains an alpha-helical coil with an unexplored biological function in osteosarcoma. This study investigated the expression of RILP in osteosarcoma cells and tissues to determine the effect of RILP on the biological behaviors of osteosarcoma cells and the underlying mechanism.

**Methods:**

Tumor Immune Estimation Resource (TIMER) database, The Cancer Genome Atlas (TCGA) database and Gene Expression Omnibus (GEO) database were used for bioinformatic analysis. Co-immunoprecipitation experiment was used to determine whether the two proteins were interacting. In functional tests, cell counting kit-8 (CCK-8) assay, colony formation assay, wound healing assay, transwell invasion assay, Immunofluorescence (IF) assay and immunohistochemical (IHC) assay were performed.

**Results:**

Overexpression of RILP significantly inhibited proliferation and impaired metastasis ability of osteosarcoma cells, while silencing of RILP showed the opposite trend. RNA-seq data analysis was applied in 143B cells and pathway enrichment analysis revealed that differentially expressed genes were mainly enriched in the PI3K/AKT pathway. We further verified that overexpression of RILP restrained the PI3K/AKT/mTOR signaling pathway and induced autophagy in osteosarcoma cells, while the opposite trend was observed when PI3K pathway activator 740Y-P was used. 3-Methyladenine (3-MA), a selective autophagy inhibitor, partially attenuated the inhibitory effect of RILP on the migration and invasion ability of osteosarcoma cells, suggesting the involvement of autophagy in epithelial–mesenchymal transition regulation in osteosarcoma cells. Growth factor receptor binding protein-10 (Grb10), an adaptor protein, was confirmed as a potential target of RILP to restrain the PI3K/AKT signaling pathway. We subcutaneously injected stably overexpressing 143B osteosarcoma cells into nude mice and observed that overexpression of RILP inhibited tumor growth by inhibiting the PI3K/AKT/mTOR pathway.

**Conclusion:**

Our study revealed that the expression of RILP was associated with favorable prognosis of osteosarcoma and RILP inhibits proliferation, migration, and invasion and promotes autophagy in osteosarcoma cells via Grb10-mediated inhibition of the PI3K/AKT/mTOR signaling pathway. In the future, targeting RILP may be a potential strategy for osteosarcoma treatment.

**Supplementary Information:**

The online version contains supplementary material available at 10.1186/s10020-023-00722-6.

## Introduction

Osteosarcoma, the most prevalent malignant bone tumor, frequently affects teenagers and has a 5-year survival rate of fewer than 20% (Shoaib et al. 2022). Early manifestations mainly include local pain and swelling, and it is prone to fracture and lung metastasis in the advanced stages (Gianferante et al. 2017). Currently, the main treatment method is neoadjuvant chemotherapy, which includes preoperative chemotherapy, intraoperative radical resection, and postoperative chemotherapy (Whelan and Davis 2018). Although neoadjuvant chemotherapy has improved the long-term survival rate of patients with localized osteosarcoma from 20% to 65**–**70%, approximately 15**–**20% of patients are diagnosed with clinical metastasis, with patients developing distant metastasis having a poor prognosis, especially lung metastasis (Isakoff et al. 2015; Wang et al. 2020a, b). Based on the above difficulties in clinical treatment, it is urgent to understand the specific regulatory mechanisms related to osteosarcoma proliferation and invasion to identify important targets for treating osteosarcoma.

Rab-interacting lysosomal protein (RILP) contains an alpha-helical coil involved in a series of physiological processes, such as autophagosome biogenesis, transport, and degradation. RILP interacts with activated Rab7 through its carboxyl terminal region, thereby driving late endosome/lysosome transport, especially lysosomal localization. (Khobrekar and Vallee 2020; Amaya et al. 2016). In recent years, RILP has been shown to play an important role in a variety of tumors. For example, Wang et al. (2015) suggested that RILP as a therapeutic target against breast cancer, and that hyper-expression of RILP suppressed the proliferation, migration, and invasion of breast cancer by inhibiting the interaction of Ras-like proto-oncogene A (RalA) and Ral guanine nucleotide dissociation stimulator (RalGDS). Lin et al. (2021) concluded that RILP acts as a tumor suppressor in lung cancer and that methylation of RILP can significantly promote the progression of lung cancer. In hepatocellular carcinoma, Rab7-RILP-regulated lysosomal trafficking is an important factor in regulating the invasive ability of cancer cells (Qi et al. 2022). These studies shown that RILP might be a good therapeutic target in various tumors; however, its specific mechanism in osteosarcoma remains unclear.

In this study, we investigated the unique role of RILP on proliferation, migration, invasion and autophagy of osteosarcoma using osteosarcoma cell lines and animal models. Our work presents a novel therapeutic approach for the treatment of osteosarcoma and highlights the vital role of RILP as a therapeutic target in osteosarcoma treatment.

## Materials and methods

### Sample collection

From 2020 to 2022, twelve groups of human normal tissues and osteosarcoma tissues were acquired from Renmin Hospital of Wuhan University. During surgery, the human samples were taken out and put in liquid nitrogen for further testing. The acquisition and use of human samples were approved by the Ethics Committee of the Renmin Hospital of Wuhan University.

### Bioinformatic analysis

The Tumor Immune Estimation Resource (TIMER) database (https://cistrome.shinyapps.io/timer/) was used to compare the expression levels of RILP in various tumors. To evaluate the prognostic ability of RILP in patients with osteosarcoma, human samples downloaded from The Cancer Genome Atlas (TCGA) database (https://portal.gdc.cancer.gov/) were used. GSE21257 and GSE39055 were obtained from the Gene Expression Omnibus (GEO) database. Autodock Tools 1.5.7 and Pymol 2.5.4 software were used for molecular docking.

### Cell culture and transfection

Human 143B and U2OS osteosarcoma cells were purchased from Procell Life Science and Technology (Wuhan, China). Osteosarcoma cells were cultured in RPMI-1640 medium (Gibco, USA) supplemented with 10% fetal bovine serum (Gibco, USA) and were incubated with 5% CO_2_ at 37 °C. For lentivirus transfection, we constructed RILP overexpression (LV-RILP), RILP knockdown (shRILP), and empty control (LV-Control and shNC) using a lentivirus obtained from OBiO (Shanghai, China). 5 µg/mL puromycin were used to screen out stably constructed osteosarcoma cells (Yang et al. 2023). In addition, 740Y-P (30 µg/mL) (Wang et al. 2020a, b) and 3-methyladenine (3-MA) (5 mM) (Lei et al. 2021) were purchased from MedChemExpress (USA).

### Cell counting kit-8 (CCK-8) and colony formation assay

Briefly, for CCK-8 assays, the cells were evenly plated in a 96-well plate and cultured for 1, 2, 3, 4 and 5 days, followed by the addition of 10 µL of CCK-8 reagent to each well. A microplate reader (Thermo Fisher, USA) was applied to determine the viability of cells by detecting the OD450 (optical density) of each well. For colony formation assays, 5 × 10^2^ cells from the four groups (LV-Control, LV-RILP, shNC, and shRILP) were evenly plated into a 6-well plate. After the cells were cultured for 2 weeks, the colonies were fixed with 4% paraformaldehyde and then stained with 1% crystal violet. Finally, cell proliferation ability was measured by counting the number of colonies.

### Wound healing assay

Initially, a sterile pipette tip with a volume of 1 ml was used to gently run across the cell surface when the cell density reached 70%. Subsequently, the treated cells were cultured in serum-free medium for 24 h. The wound area was observed under an inverted microscope (Olympus, Japan) and measured using ImageJ software.

### Transwell invasion assay

Invasion experiments were conducted using Transwell chambers (Corning, USA) and Matrigel (Corning, USA). In the upper chamber, medium was introduced at a ratio of 1:6, followed by medium containing 20% FBS in the bottom chamber. After complete solidification, 200 µL of medium containing 1 × 10^5^ cells were added to the upper chamber. After incubation with 5% CO_2_ at 37 °C for 48 h, the upper chamber was fixed with 4% paraformaldehyde and stained with 1% crystal violet. The invading cells from the upper chamber were observed under an inverted microscope (Olympus, Japan) and measured using ImageJ software.

### Western blot analysis

Total protein was extracted from cells and tissues using radio-immunoprecipitation assay (RIPA) buffer (Servicebio Technology, Wuhan, China). A bicinchoninic acid (BCA) kit (Servicebio Technology, Wuhan, China) was used to quantify the protein concentration. Then total protein was separated by electrophoresis and transferred to a membrane. After blocking and washed three times with tris-buffered saline in tween (TBST), the membrane was incubated with primary antibodies overnight at 4 °C. Subsequently, the membrane was washed and incubated with the secondary antibodies. Finally, bands were visualized using an enhanced chemiluminescence (ECL) kit (Servicebio, Wuhan, China).

### Immunoprecipitation (IP)

143B and 293T cells were collected, and IP lysate (50 mM Tris HCl, 1 mM EDTA, 150 mM NaCl) was added. Subsequently, protein A/G beads were added followed by antibodies and incubated while shaking at 4 °C overnight. On the following day, the protein A/G beads were collected and washed three times with IP buffer, then the bound proteins were heated in a metal bath at 95 °C and detected using western blot assay.

### Animal studies

All of our experiments were authorized by the Ethics Committee of the Renmin Hospital of Wuhan University. Male nude mice (4–5 weeks old) were purchased from Shulaibao Biotech (Wuhan, China). The mice were randomly divided into two groups of six mice each (LV-Control, LV-RILP) and then subcutaneously injected with 5 × 10^6^ stably transfected 143B osteosarcoma cells. The mice were then kept in a standard experimental environment supplemented with food and water and observed weekly for tumor size and volume. Before sampling, all nude mice were euthanized with 2% pentobarbital sodium (150 mg/kg) (Mohamed et al. 2020; Xie et al. 2022). Four weeks after molding, all mice were sacrificed in peace and the tumors were weighed. Finally, all tumors were placed in liquid nitrogen or fixed in 4% paraformaldehyde for further preservation.

### Immunohistochemistry (IHC) staining

After fixing with 4% paraformaldehyde for 24 h, the tumor tissue was wrapped with wax and cut into 4-µm slices. Then the slices were blocked with 1% bovine serum albumin. After these slices were incubated with primary and secondary antibodies, a DAB (diaminobenzidine tetrahydrochloride) kit (CST, USA) was used for chromogenic detection.

### Statistical analysis

All data are presented as mean ± standard deviation (SD). SPSS 6.0 and GraphPad Prism 8.0 was used for statistical analysis. Each experiment was repeated three times. Differences were considered statistically significant at *P* < 0.05.

## Results

### RILP is significantly downregulated in osteosarcoma tissues, and its lower level predicts poor prognosis in osteosarcoma patients

**Fig. 1:**
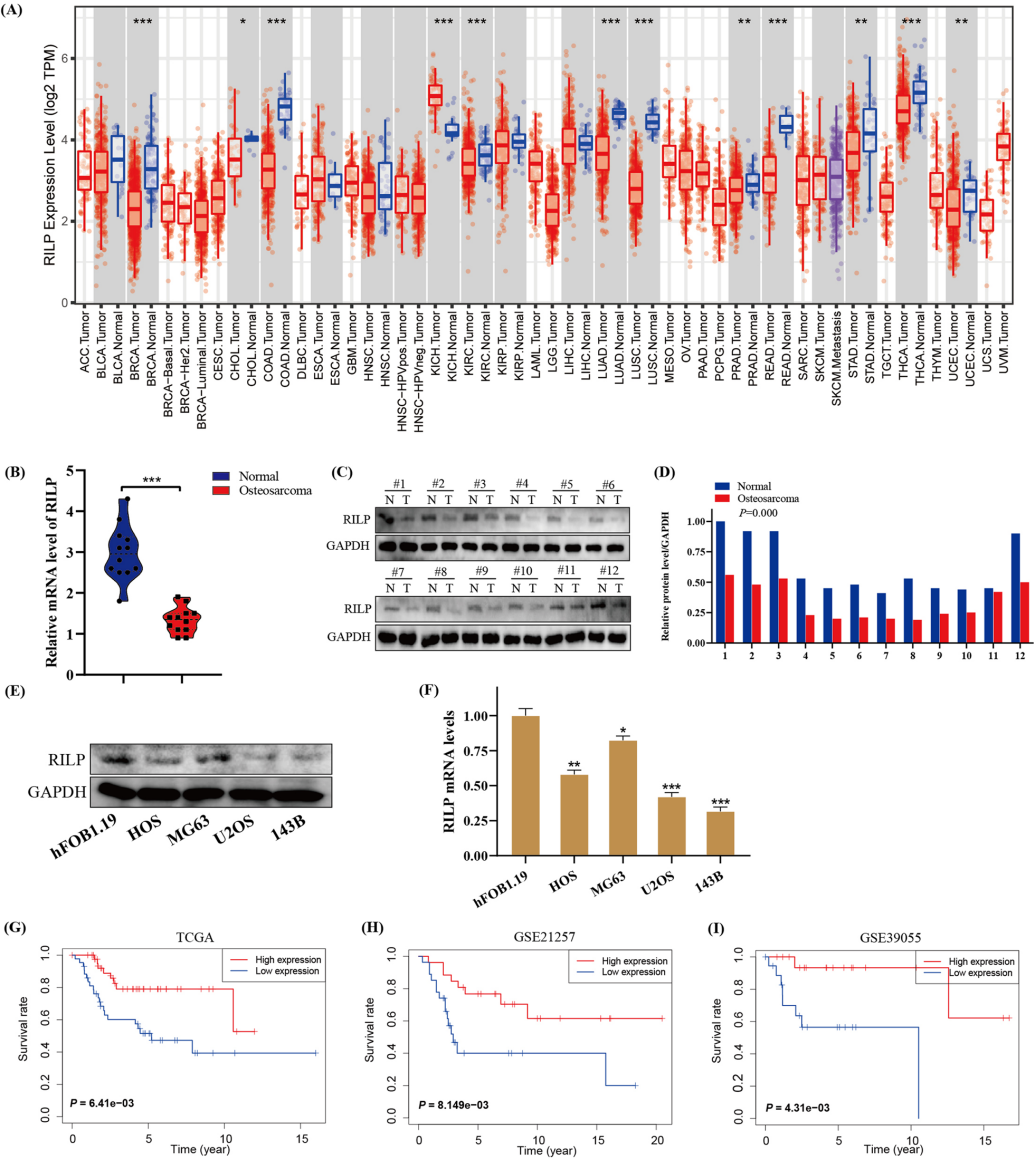
RILP is downregulated in osteosarcoma and its lower level correlates with poor prognosis in osteosarcoma patients. **A** Expression profile of RILP in various tumors based on TIMER database. **B** The relative mRNA level of RILP in tumor and adjacent tissues based on clinical samples. **C** The protein expression level of RILP in tumor and adjacent tissues on clinical samples was detected by western blotting. GAPDH was used as a loading control. **D** Quantitative analysis of the protein expression level of RILP. **E** The protein expression level of RILP in human osteoblasts (hFOB1.19) and osteosarcoma cell lines (HOS, MG63, U2OS and 143B) were subjected to western blotting analysis. GAPDH was used as a loading control. **F** The relative mRNA level of RILP in human osteoblasts (hFOB1.19) and osteosarcoma cell lines (HOS, MG63, U2OS and 143B). **G**–**I** Kaplan–Meier survival analysis of osteosarcoma patients with high and low expression of RILP in TCGA and GEO database. *P < 0.05, **P < 0.01, ***P < 0.001

Using the TIMER database, we found that RILP was significantly differentially expressed in several cancers (Fig. 1A). We then measured the expression level of RILP in human osteosarcoma and normal tissue specimens. The protein and mRNA expression of RILP in osteosarcoma specimens was lower than those in adjacent normal tissues (Fig. 1B–D). In addition, the protein and mRNA expression levels of RILP at the cellular level revealed that osteosarcoma cells expressed lower level of RILP than that in hFOB1.19 cells, especially in 143B and U2OS cells (Fig. 1E, F). Finally, Kaplan-Meier survival analysis was performed using data from the GEO and TCGA databases; the results suggested that lower expression of RILP predicted poorer prognosis in patients with osteosarcoma (Fig. 1G–I).

### Overexpression of RILP inhibits the proliferation, migration, and invasion of osteosarcoma cells

**Fig. 2:**
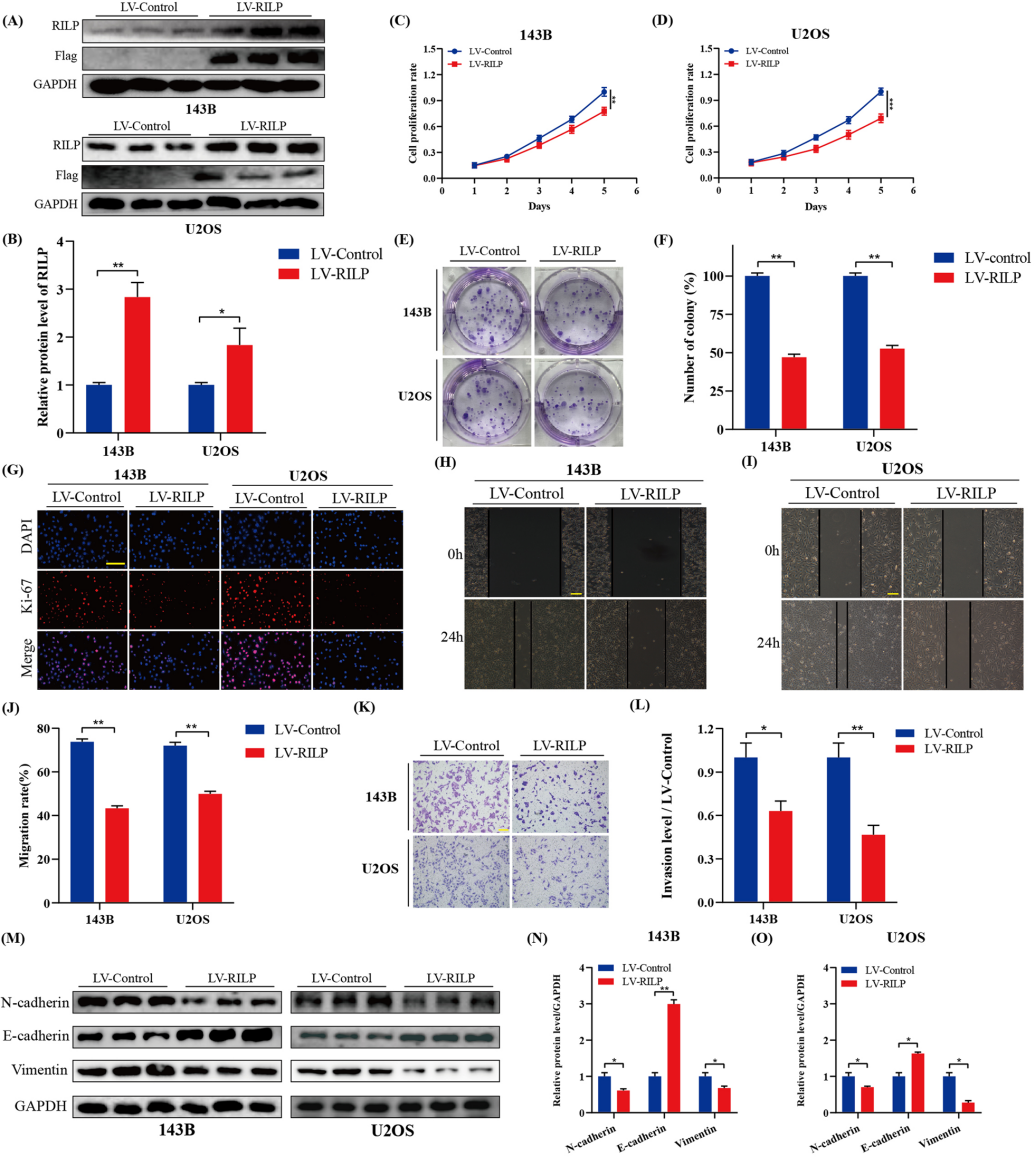
Overexpression of RILP inhibits proliferation, migration and invasion of osteosarcoma cells. **A**, **B** Overexpression of RILP was determined by western blot assays. GAPDH was used as a loading control. **C**, **D** The proliferation level of osteosarcoma after transfected with LV-RILP or LV-Control was evaluated by CCK-8 assay. **E**, **F** The colony formation ability of osteosarcoma cells stably overexpressing RILP or not was measured by colony formation assay. **G** Immunofluorescence staining assay of Ki-67 in osteosarcoma cells was performed the to measure the proliferation level, scale bar: 200 μm. **H**–**J** Representative images and quantitative analysis of osteosarcoma cell migration based on wound healing assay, scale bar: 200 μm. **K**, **L** Increased expression of RILP restrained osteosarcoma cell invasion ability based on transwell assay, scale bar: 400 μm. **M**–**O** Western blot and quantitative analyses of E-cadherin, N-cadherin and Vimentin. GAPDH was used as a loading control. *P < 0.05, **P < 0.01, ***P < 0.001

We first constructed RILP-overexpressing cell line with recombinant lentivirus, and verified RILP overexpression using western blotting (Fig. 2A, B). In Fig. 2C–F, cell proliferation rate and colony numbers were significantly lower in cells transfected with LV-RILP. As shown in Fig. 2G, the expression of Ki-67 was significantly decreased in RILP-upregulated cell lines compared with that in the control group. Subsequently, wound healing assays suggested that stable overexpression of RILP significantly impaired migration of osteosarcoma cells (Fig. 2H–J). Similarly, transwell assay revealed that RILP overexpression attenuated invasion of osteosarcoma cells (Fig. 2K–L). We also detected epithelial–mesenchymal transition (EMT)-related proteins (Fig. 2M–O), and found that the expression levels of Vimentin and N-cadherin were significantly decreased, while that of E-cadherin was increased; thus suggesting that RILP overexpression significantly restrains the metastatic potential of osteosarcoma cells.

### Silencing of RILP promotes the proliferation, migration, and invasion of osteosarcoma cells

**Fig. 3:**
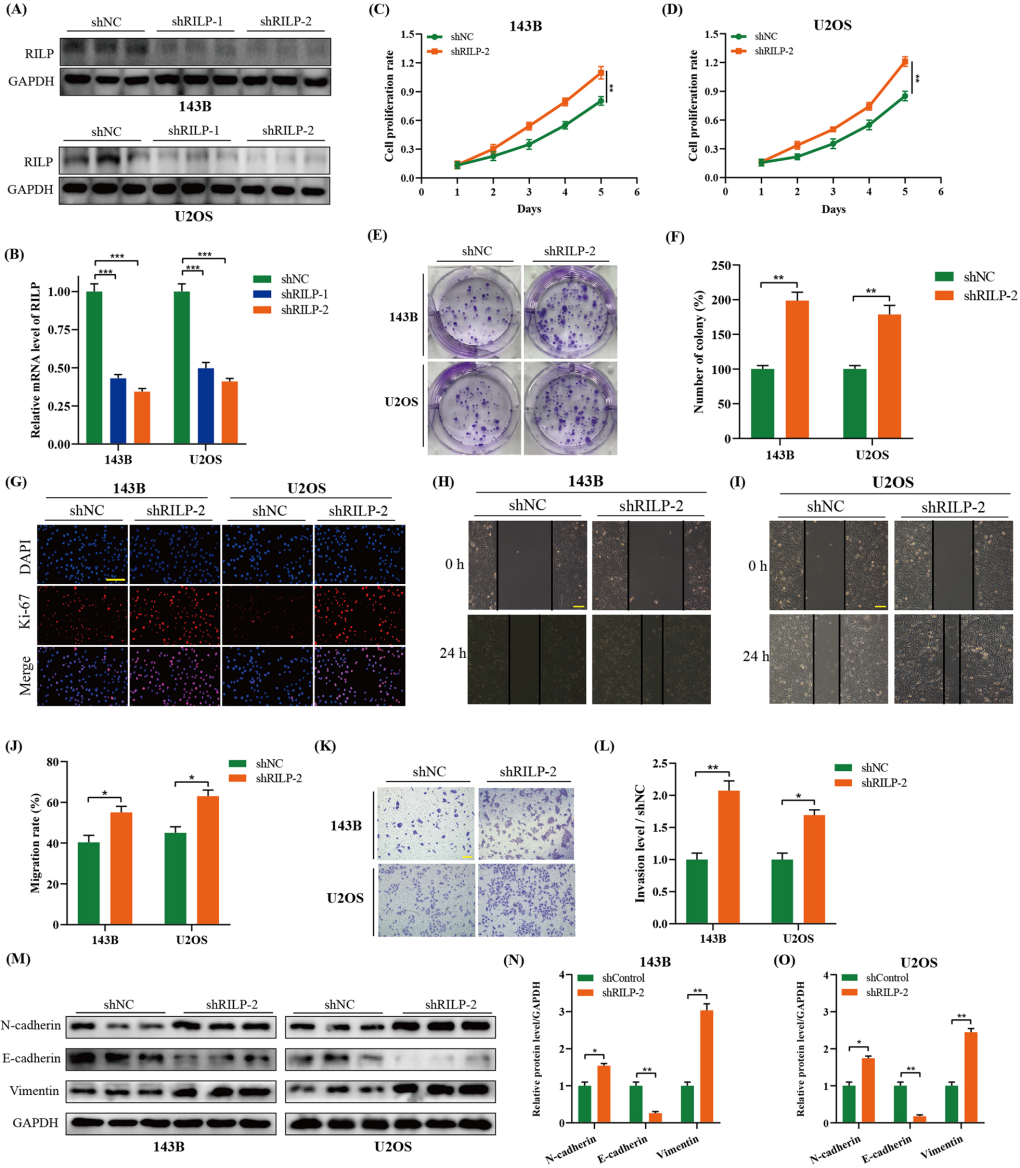
Silencing of RILP promotes proliferation, migration and invasion of osteosarcoma cells. **A**, **B** The protein expression level of RILP after transfected with shRILP or not were detected by western blot assays. GAPDH was used as a loading control. **C**, **D** CCK-8 assay was applied to measure the proliferation level of osteosarcoma cells after transfected with shRILP or not. **E**, **F** Colony formation assay was performed to evaluate the colony formation ability of osteosarcoma cells after transfected with shRILP or not. **G** Immunofluorescence staining assay of Ki-67 in osteosarcoma cells was performed the to measure the proliferation level, scale bar: 200 μm. **H**–**J** The migration ability of osteosarcoma cells stably knocking down of RILP or not was detected by wound healing assay, scale bar: 200 μm. **K**, **L** The invasion ability of osteosarcoma cells stably knocking down of RILP or not was detected by transwell assay, scale bar: 400 μm. **M**–**O** Western blot and quantitative analyses of EMT-related protein including N-cadherin, E-cadherin and Vimentin. GAPDH was used as a loading control. *P < 0.05, **P < 0.01, ***P < 0.001

Later, we also silenced RILP in osteosarcoma cells through lentivirus infection. Transfection efficiency was determined using western blotting (Fig. 3A, B). CCK-8, colony formation, and Ki-67 staining assays showed that knockdown of RILP significantly enhanced the OD450 value, colony cells, and Ki-67-positive cells, suggesting that RILP downregulation accelerates the proliferation of osteosarcoma cells (Fig. 3C–G). Wound healing and transwell assays revealed enhanced migration and invasion abilities in RILP knockout osteosarcoma cell lines (Fig. 3H–L). Western blot analysis showed increased protein expression levels of N-cadherin and Vimentin and a decreased level of E-cadherin (Fig. 3M–O); thus suggesting that the downregulation of RILP significantly promotes the metastatic ability of osteosarcoma cells.

### RILP negatively regulates the PI3K/AKT/mTOR pathway to restrain the proliferation, migration, and invasion of osteosarcoma cells

**Fig. 4:**
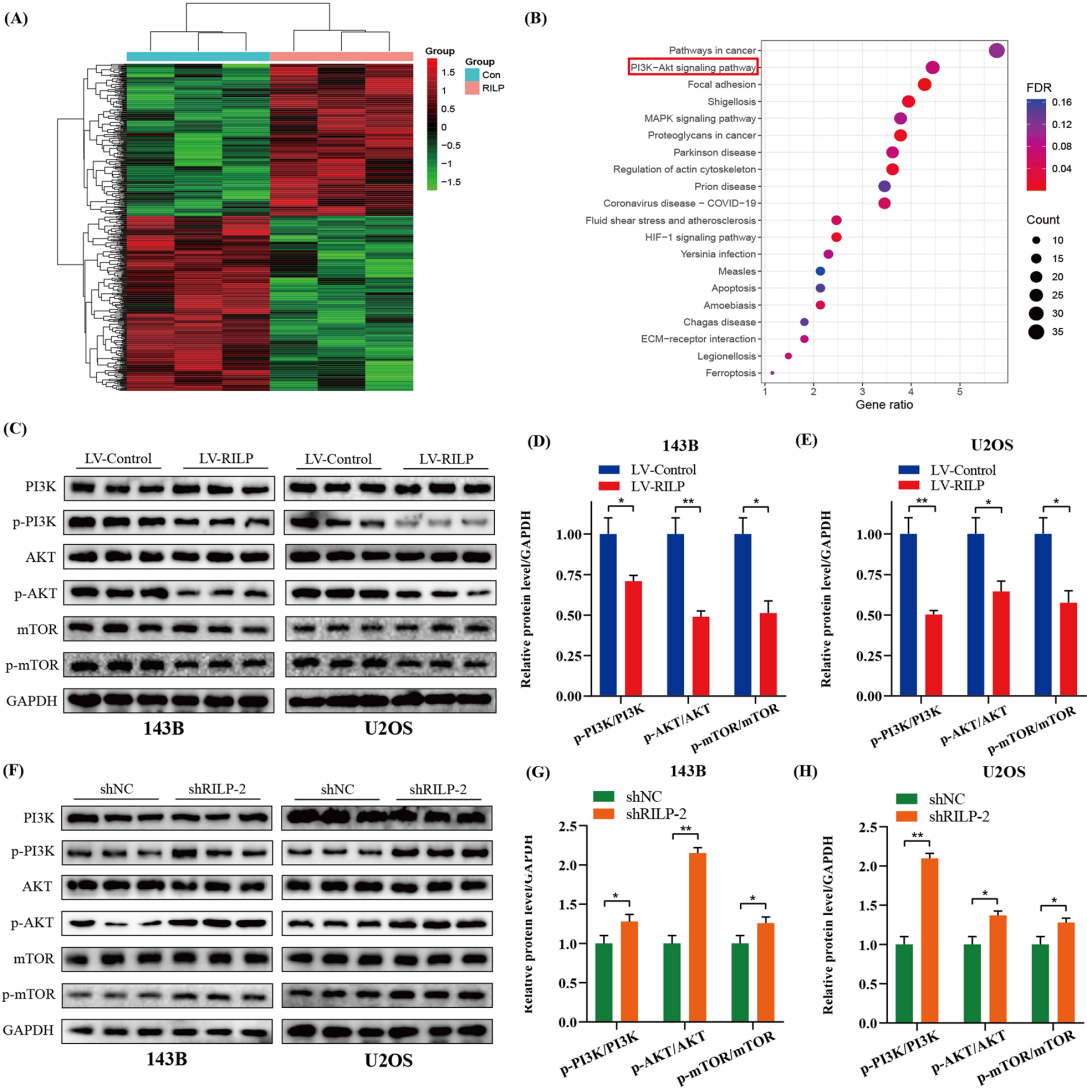
RILP negatively regulates PI3K/AKT/mTOR pathway to restrain the proliferation, migration and invasion of osteosarcoma cells. **A** Heatmap illustrating RILP-related genes based on difference analysis. **B** KEGG enrichment analysis revealed that RILP-associated genes are mainly enriched in PI3K-AKT signaling pathway. **C**–**E** Western blot assay was performed to detect the protein expression level of PI3K, p-PI3K, AKT, p-AKT, mTOR and p-m-TOR in osteosarcoma cells stably overexpressing RILP. GAPDH was used as a loading control. **F**–**H** Western blot assay was performed to detect the protein expression level of PI3K, p-PI3K, AKT, p-AKT, mTOR and p-m-TOR in osteosarcoma cells stably knocking down RILP. GAPDH was used as a loading control. *P < 0.05, **P < 0.01

RNA-seq analysis was performed on 143B cells with or without RILP overexpression. Genes that were upregulated or downregulated after RILP overexpression are shown in Fig. 4A. KEGG enrichment analysis showed that differentially expressed genes were mainly enriched in the PI3K/AKT pathway (Fig. 4B). We found the overexpression of RILP significantly decreased the protein levels of p-PI3K, p-AKT, and p-mTOR (Fig. 4C–E). Conversely, RILP knockdown increased the protein expression of p-PI3K, p-AKT, and p-mTOR (Fig. 4F–H). Subsequently, the cells were treated with 740Y-P, a highly selective activator of PI3K, and partial reversal of the inhibitory effects of RILP on the proliferation, migration, and invasion of osteosarcoma cells, were observed (Additional file 1: Fig. S1A–F). These results suggest that RILP negatively regulates the PI3K/AKT/mTOR pathway in osteosarcoma cells.

### RILP induces osteosarcoma cell autophagy by inhibiting the PI3K/AKT/mTOR pathway

**Fig. 5:**
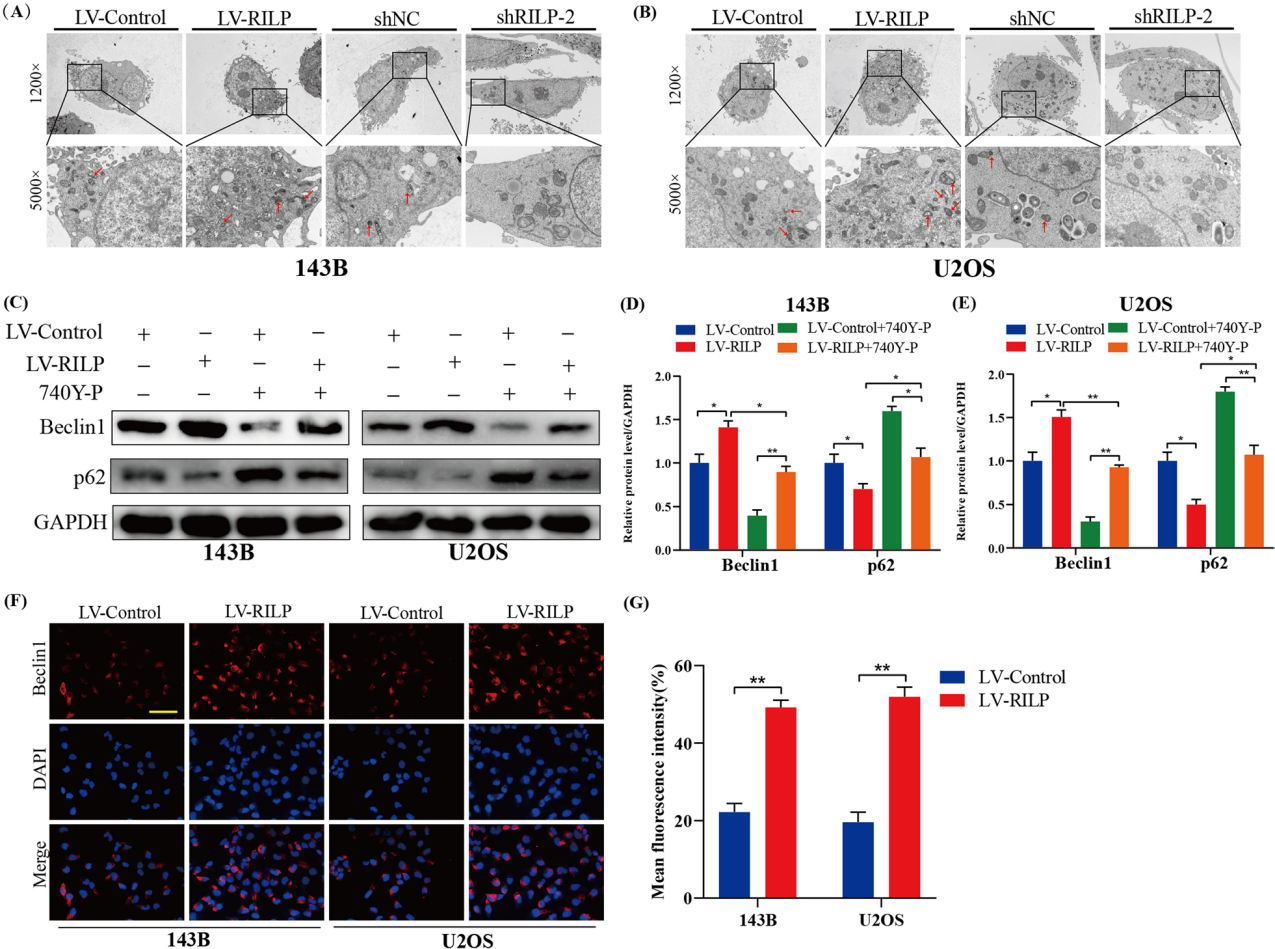
RILP induces OS cell autophagy by inhibiting PI3K/AKT/mTOR pathway. **A**, **B** Representative transmission electron micrographs autophagic vesicles in 143B and U2OS cells stably transfected with shNC/shRILP and LV-Control/LV-RILP. **C**–**E** The protein expression level of Beclin1 and p62 in osteosarcoma cells was detected after treated with 740Y-P or not. GAPDH was used as a loading control. **F**, **G** Representative images and quantitative analysis of Beclin1 immunofluorescence intensity in osteosarcoma cells stably transfected with RILP or not, scale bar: 200 μm. *P < 0.05, **P < 0.01

Subsequently, we assessed the number of autophagosomes in the various groups of cells (LV-Control, LV-RILP, shNC, and shRILP) using transmission electron microscopy (Fig. 5A, B). Compared with LV-Control, the number of autophagosomes in the RILP-overexpression group was the highest, whereas only a few autophagosomes were observed in the RILP-knockdown group. Meanwhile, the expression levels of autophagy-related proteins in osteosarcoma cells were also measured (Fig. 5C–E). Consistent with the immunofluorescence results (Fig. 5F, G), we found that the expression level of Beclin1 was significantly increased in the RILP-overexpression group, while p62 was significantly decreased. Finally, cells were treated with 740Y-P; we found that the activation of autophagy mediated by RILP upregulation was significantly attenuated when treated with 740Y-P (Fig. 5C–E). Our results demonstrated that RILP was involved in the regulation of autophagy via inhibition of the PI3K/AKT/mTOR pathway.

### Effect of RILP on the metastasis capacities of osteosarcoma cells is mediated by autophagy

**Fig. 6:**
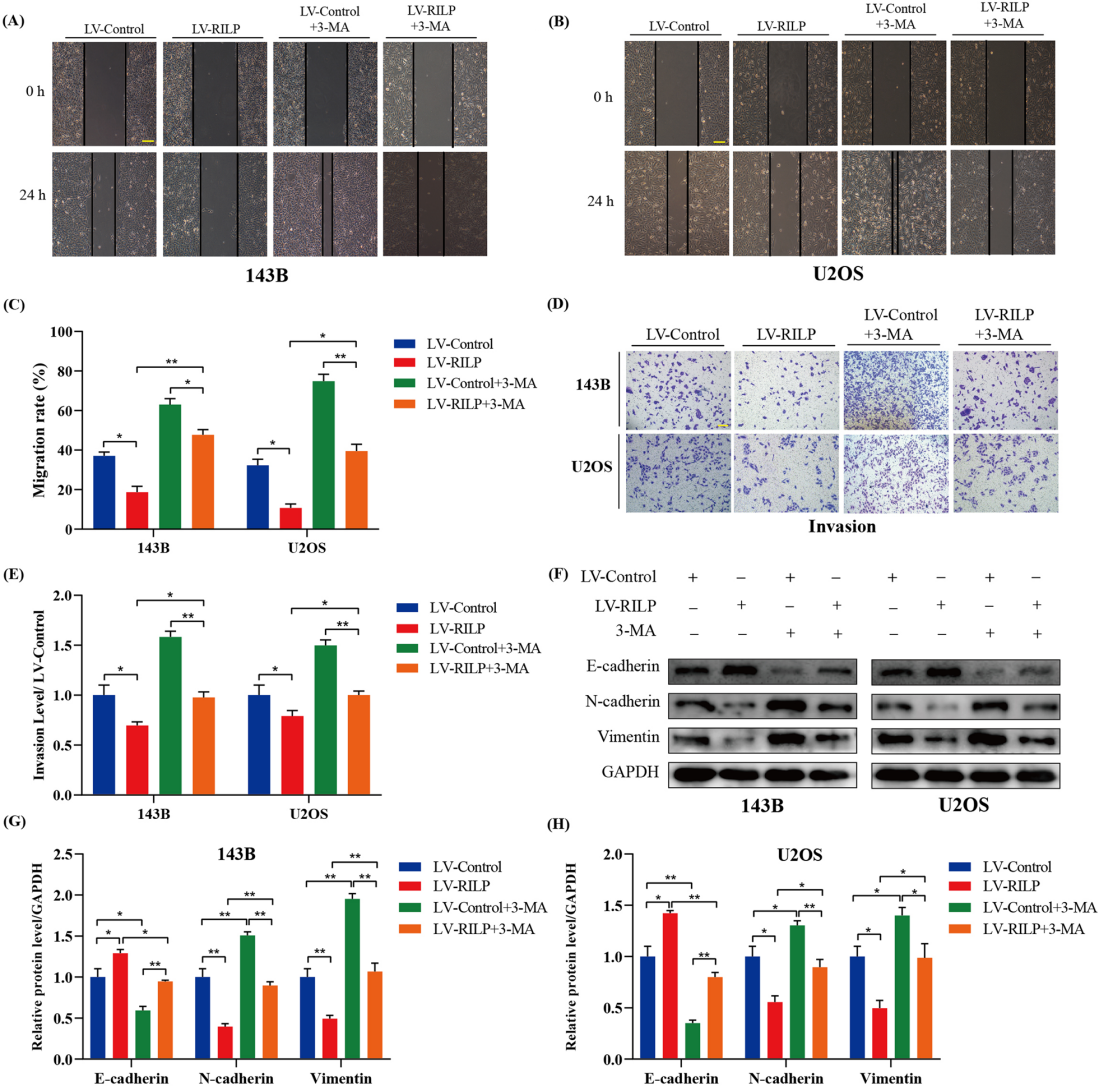
The effect of RILP expression can affect migration and invasion of osteosarcoma cells that is mediated by autophagy. **A**–**C** Wound healing assay comparing the motility of osteosarcoma cells after treated with 3-methyladenine or not, scale bar: 200 μm. **D**, **E** Transwell invasion assay was performed to compare the invasion ability of osteosarcoma cells after treated with 3-methyladenine or not, scale bar: 400 μm. **F**–**H** Western blot analysis was applied to measure the protein expression level of E-cadherin, N-cadherin and Vimentin. GAPDH was used as a loading control. *P < 0.05, **P < 0.01

3-MA, an autophagy inhibitor, was used in this study. In the wound healing and transwell invasion assays, 3-MA treatment attenuated the inhibitory effect of RILP on the metastasis ability of osteosarcoma cells (Fig. 6A–E). Subsequently, western blotting results suggested that 3-MA treatment impaired the effect of RILP overexpression-induced upregulation of E-cadherin and downregulation of N-cadherin and Vimentin (Fig. 6F–H). Thus, our results suggested that the overexpression of RILP increased the autophagy level of osteosarcoma cells, thereby inhibiting osteosarcoma cell migration and invasion.

### RILP restrains osteosarcoma progression via Grb10-mediated inhibition of the PI3K/AKT/mTOR pathway

**Fig. 7:**
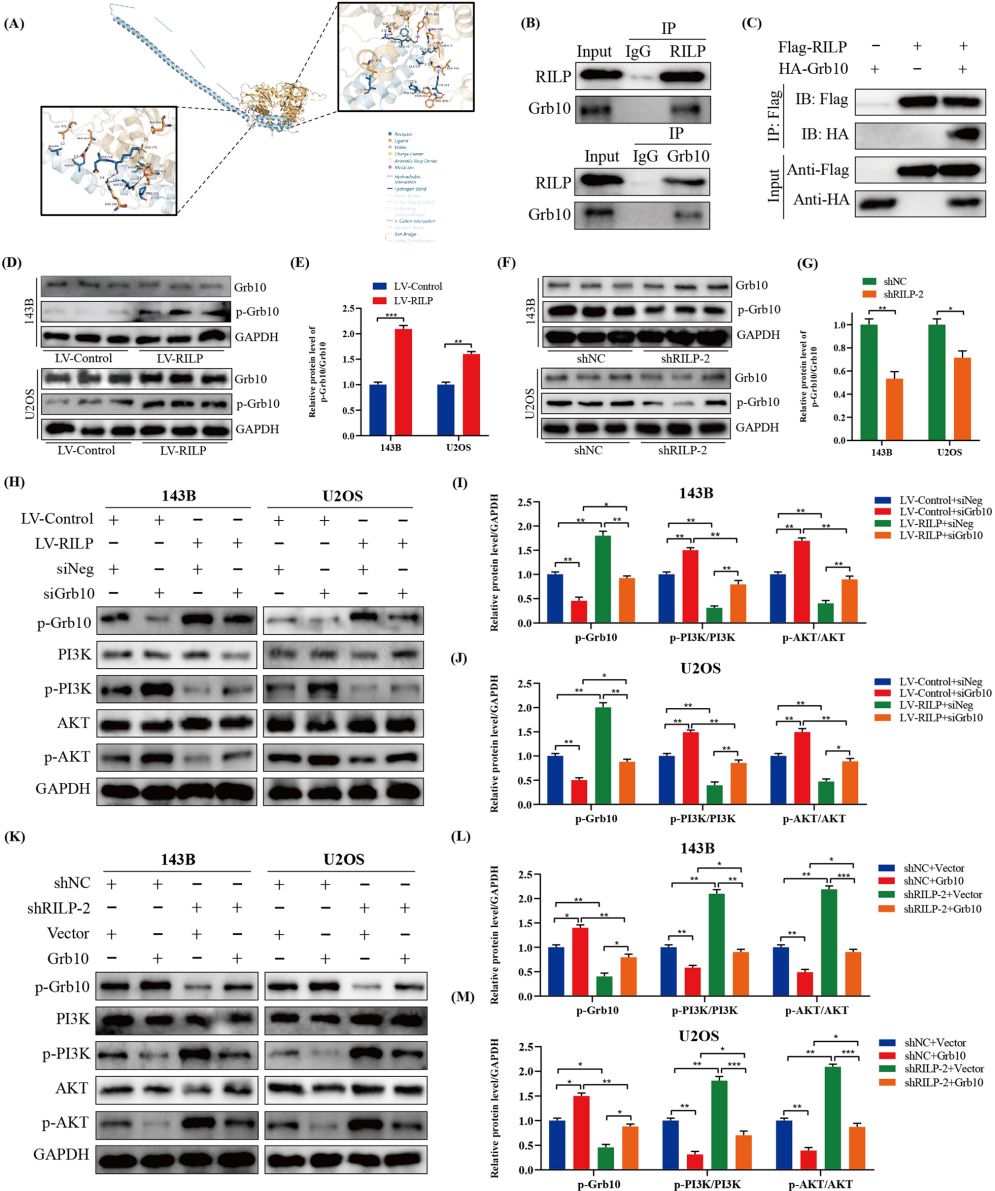
RILP restrains osteosarcoma progression via Grb10-mediated inhibition of PI3K/AKT/mTOR pathway. **A** Molecular docking techniques show that RILP can interact with Grb10. **B** RILP and Grb10 bind endogenously in 143B cells. **C** RILP and Grb10 bind exogenously in 293T cells transfected with flag-RILP and HA-Grb10 plasmid. **D**, **E** Western blot and quantitative analyses of Grb10 and p-Grb10 in RILP-upregulated cells. **F**, **G** Western blot and quantitative analyses of Grb10 and p-Grb10 in RILP-silenced cells. **H**–**J** Evaluation of p-PI3K and p-AKT in RILP-overexpressing cells after transfection of siGrb10 or siNeg. **K**–**M** Evaluation of p-PI3K and p-AKT in RILP-silenced after transfected with Grb10 overexpression plasmid or empty vector. *P < 0.05, **P < 0.01, ***P < 0.001

Using molecular docking technology, growth factor receptor binding protein-10 (Grb10), which is involved in the PI3K/AKT pathway, was identified to interact with RILP (Fig. 7A). Co-immunoprecipitation assay in 143B osteosarcoma cells showed that RILP interacted with Grb10 (Fig. 7B). Similarly, in HEK 293T cells transfected with flag-tagged RILP plasmid and HA-tagged Grb10 plasmid, RILP co-precipitated with Grb10 (Fig. 7C). Subsequently, results showed that the expression level of phosphorylated Grb10 was significantly upregulated following overexpression of RILP (Fig. 7D, E), while knockdown showed the opposite trend (Fig. 7F, G). Thus, our results suggested that RILP regulates Grb10 by increasing its phosphorylation.

Meanwhile, as shown in Fig. 7H–J, the knockdown of Grb10 significantly attenuated the inhibition effect of RILP on the PI3K/AKT signaling pathway. Conversely, overexpression of Grb10 significantly attenuated the RILP knockdown-induced promotion of the PI3K/AKT signaling pathway (Fig. 7K–M). Functional experiments suggested that downregulation of Grb10 attenuated the inhibition effect of RILP on osteosarcoma cell proliferation, migration, and invasion, the opposite is true for Grb10 overexpression (Additional file 2: Fig. S2A–H). In conclude, RILP restrains osteosarcoma progression via Grb10-mediated inhibition of the PI3K/AKT/mTOR pathway.

### Increased expression of RILP inhibits tumor growth in vivo

**Fig. 8:**
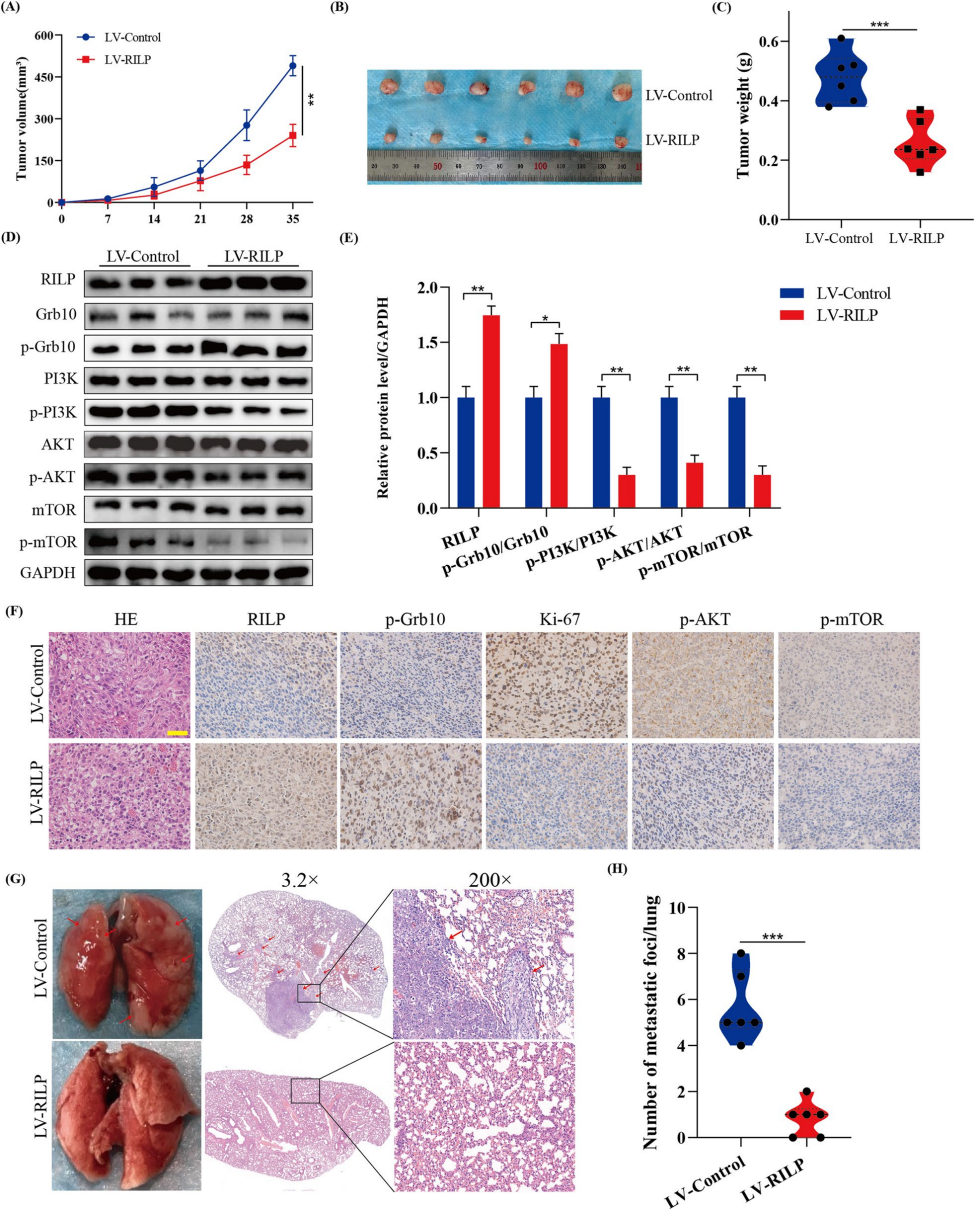
Increased expression of RILP inhibits tumor growth in vivo. **A** Tumor volume was monitored every 7 days to measure the tumor growth in vivo. **B** Comparison of xenograft tumor size in nude mice after injecting 143B osteosarcoma cells stably transfected with LV-Control/LV-RILP. **C** Tumor weight was compared between two groups. **D**, **E** Western blot and quantitative analyses of RILP, Grb10, p-Grb10, PI3K, p-PI3K, AKT, p-AKT, mTOR and p-mTOR in xenograft tumor. **F** IHC analysis of RILP, p-Grb10, Ki-67, p-AKT, p-mTOR for tissues of xenograft tumors. Scale bar: 200 μm. **G**, **H** Representative H&E staining images of lung tissue in two groups, the red arrows represent lung metastasis. *P < 0.05, **P < 0.01, ***P < 0.001

We subcutaneously injected 143B osteosarcoma cells stably expressing RILP and control cells into nude mice, and the tumor volume was monitored every 7 days. As shown in Fig. 8A–C, compared with LV-Control, the tumors induced by RILP-overexpressing 143B osteosarcoma cells were much smaller, suggesting that RILP upregulation reduced tumor size in vivo*.* Moreover, the protein expression levels of p-PI3K, p-AKT, and p-mTOR in the RILP overexpression group were significantly lower than those in the control group, as confirmed using western blotting and IHC (Fig. 8D–F). In addition, IHC results suggested a decreased level of Ki-67 in the RILP overexpression group compared with that in the control group (Fig. 8F). Finally, we identified the inhibitory effect of RILP overexpression on tumor metastasis in vivo by injecting stable cells (LV-Control and LV-RILP) into the mouse tail vein, and found that the number of lung metastasis in the RILP-overexpression group was significantly reduced compared with that of the control group (Fig. 8G, H).

## Discussion

RILP has been widely studied due to its unique role in various biological processes, such as autophagosome biogenesis, transport, and degradation (Khobrekar and Vallee 2020; Sapmaz et al. 2019; Khobrekar et al. 2020). Recently, an increasing number of researches have also associated the regulation of autophagy by RILP in tumors (Qi et al. 2022; Luca et al. 2021; Steffan et al. 2010). RILP is a Rab7 adaptor protein responsible for linking Rab7-containing vesicles to dynein motor complexes to drive microtubule minus-end-directed transport toward the microtubule organizing center. Although RILP has been reported to regulate biological processes in many tumors, such as prostate cancer, breast cancer, and hepatocellular carcinoma, its role in osteosarcoma has not been clarified until now.

Using a large independent database and human tissue samples, we systematically analyzed the differences in the expression of RILP between tumor and normal tissues and confirmed that RILP was significantly decreased in tumor tissues compared with that in normal tissues. Clinically, osteosarcoma patients with higher expression levels of RILP have a better outcome than those with lower expression levels. Additionally, upregulation of RILP restrained the progression of osteosarcoma cells. Conversely, silencing of RILP promoted the malignancy of osteosarcoma cells, further demonstrating the tumor suppressor function of RILP. Therefore, our results validated that RILP was a tumor suppressor gene and a potential prognostic factor for osteosarcoma.

The PI3K/AKT/mTOR signaling pathway is one of the most critical intracellular signaling pathways controlling basic cellular functions, including cell proliferation, survival, metabolism, and locomotion (Yu et al. 2021; Corti et al. 2019). Using RNA-seq data analysis, we uncovered the mechanism of action of RILP in osteosarcoma, at least in part, through its regulation of the PI3K/AKT/mTOR pathway, which in turn regulated the proliferation, migration, and invasion of osteosarcoma cells. 740Y-P, a highly selective activator of PI3K, was found to attenuate the inhibitory effect on osteosarcoma cell proliferation, migration, and invasion caused by RILP overexpression. This further suggests that the PI3K/AKT/mTOR signaling pathway plays a key role in cancer inhibition mediated by RILP.

Autophagy is a highly conserved process that degrades some organelle fragments in lysosomes and is considered the second type of programmed cell death (Chen et al. 2019; Yoshida 2017). Meanwhile, the PI3K/AKT/mTOR signaling pathway also plays an important regulatory role in autophagy (Wani et al. 2019; Wang et al. 2017). Using a transmission electron microscope, we found that the number of autophagosomes were significantly upregulated in cells overexpressing RILP. In contrast, RILP knockdown reduced the number of autophagosomes. Since activation of autophagy can promote or inhibit the EMT process (Chen et al. 2019; Liu et al. 2020), we hypothesized that the effect of RILP on EMT in osteosarcoma cells is mediated through autophagy. 3-MA, a highly selective inhibitor, was applied. We found that the inhibitory effect of EMT caused by RILP overexpression could be partly reversed by 3-MA treatment. This result is consistent with the findings of Zhang et al. (2019). Overall, our research revealed that the effect of RILP on EMT in osteosarcoma cells was partly mediated through autophagy.

RILP regulates the PI3K/AKT/mTOR signaling pathway through interacting with Grb10, an adapter protein containing the Src homology 2 (SH2) domain (Liu et al. 2014). Grb10 is involved in the regulation of various biological processes, including growth, cell proliferation, and insulin signaling (Plasschaert and Bartolomei 2015). Meanwhile, phosphorylated Grb10 negatively affects the mTOR signaling in brown adipose cells (Liu et al. 2014). Tyrosine phosphorylation of Grb10 may play a key role in cell signal transduction mediated by Src tyrosine kinase (Langlais et al. 2000). However, the specific role of Grb10 in osteosarcoma remains nebulous. In this study, we observed that RILP could interact with Grb10 and promote its phosphorylation, thus affecting the malignant phenotypes of osteosarcoma. Since Grb10 is involved in negative regulation of the PI3K/AKT signaling pathway (Hsu et al. 2011; Yu et al. 2011), we hypothesized that RILP could interact with Grb10 to inhibit the PI3K signaling pathway, ultimately affecting the osteosarcoma phenotype. Therefore, we changed the expression levels of RILP and Grb10, respectively, and observed the changes in related indicators of the PI3K signaling pathway. Our results identified that RILP could bind to Grb10 and participate in the inhibition of the PI3K signaling pathway, thus affecting the progression of osteosarcoma.

Consistent with the results of Lin et al. (2021) and Wang et al. (2015), RILP is also a tumor suppressor gene in osteosarcoma, and its mechanism is mainly related to PI3K signaling pathway-mediated autophagy. However, the drawback of this study is that the specific reasons for RILP downregulation in osteosarcoma were not investigated in detail. Since abnormal DNA methylation is the most common molecular lesion in cancer cells(Meng et al. 2022; Vos et al. 2017; Tyagi et al. 2017). We suspected that whether hypermethylation of promoter sites leads to a reduction of RILP expression in osteosarcoma. In addition, there are many small molecules upstream of RILP that can affect its expression level, including Rab36 (Matsui et al. 2012), Rab7 (Cantalupo et al. 2001) and Rab24 (Amaya et al. 2016). Thus, subsequent studies warrant the verification of these speculations.

## Conclusions

**Fig. 9:**
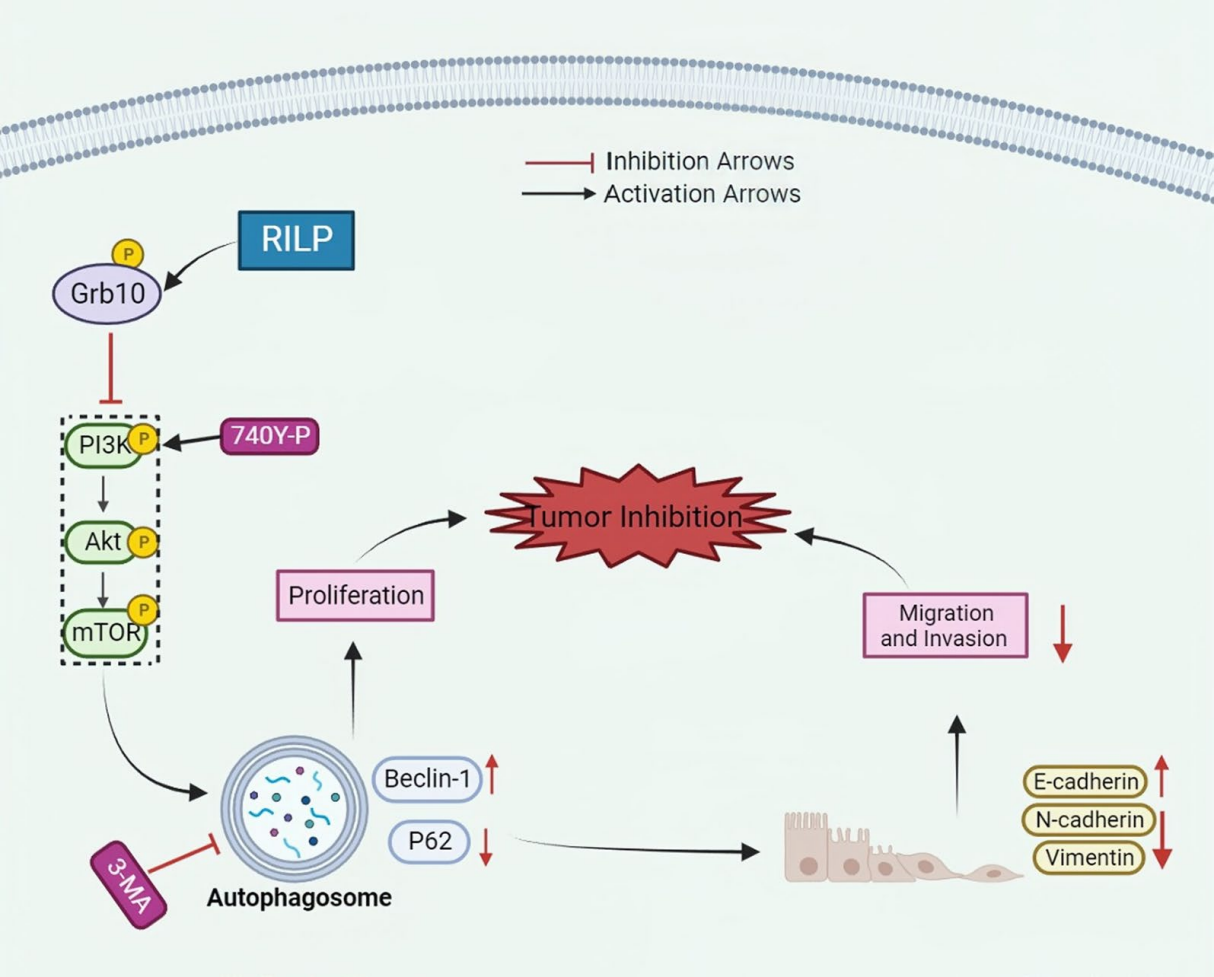
Working model of RILP regulating proliferation and autophagy in osteosarcoma

Our study demonstrated the role of RILP as a tumor suppressor gene in osteosarcoma. A higher expression of RILP is associated with lower malignancy and more favorable outcomes in osteosarcoma patients. Functionally, RILP inhibits proliferation, migration, invasion, and promotes autophagy in osteosarcoma cells via Grb10-mediated inhibition of the PI3K/AKT/mTOR signaling pathway (Fig. 9). Therefore, combined targeting of RILP and the PI3K/AKT/mTOR signaling pathway may provide a new therapeutic strategy for osteosarcoma.

### Supplementary Information

**Additional file 1: Figure S1.** (A–F) CCK-8, wound healing, and transwell invasion assays were performed to access the effects of 740Y-P (an activator of PI3K signaling pathway) on osteosarcoma cell proliferation, migration, and invasion. *P < 0.05, **P < 0.01, ***P < 0.001.

**Additional file 2: Figure S2.** (A–H) CCK-8, wound healing, and transwell invasion assays showed that Grb10 mediated the effect of RILP on osteosarcoma cell proliferation, migration, and invasion. *P < 0.05,**P < 0.01.

## Data Availability

All the data sets used and/or analyzed in our study are available.

## References

[CR1] Amaya C, Militello RD, Calligaris SD, Colombo MI (2016). Rab24 interacts with the Rab7/Rab interacting lysosomal protein complex to regulate endosomal degradation. Traffic.

[CR2] Cantalupo G, Alifano P, Roberti V, Bruni CB, Bucci C (2001). Rab-interacting lysosomal protein (RILP): the Rab7 effector required for transport to lysosomes. Embo J.

[CR3] Chen HT, Liu H, Mao MJ, Tan Y, Mo XQ, Meng XJ, Cao MT, Zhong CY, Liu Y, Shan H (2019). Crosstalk between autophagy and epithelial–mesenchymal transition and its application in cancer therapy. Mol Cancer.

[CR4] Corti F, Nichetti F, Raimondi A, Niger M, Prinzi N, Torchio M, Tamborini E, Perrone F, Pruneri G, Di Bartolomeo M (2019). Targeting the PI3K/AKT/mTOR pathway in biliary tract cancers: a review of current evidences and future perspectives. Cancer Treat Rev.

[CR5] De Luca M, Romano R, Bucci C (2021). Role of the V1G1 subunit of V-ATPase in breast cancer cell migration. Sci Rep.

[CR6] Gianferante DM, Mirabello L, Savage SA (2017). Germline and somatic genetics of osteosarcoma—connecting aetiology, biology and therapy. Nat Rev Endocrinol.

[CR7] Hsu PP, Kang SA, Rameseder J, Zhang Y, Ottina KA, Lim D, Peterson TR, Choi Y, Gray NS, Yaffe MB (2011). The mTOR-regulated phosphoproteome reveals a mechanism of mTORC1-mediated inhibition of growth factor signaling. Science.

[CR8] Isakoff MS, Bielack SS, Meltzer P, Gorlick R (2015). Osteosarcoma: current treatment and a collaborative pathway to success. J Clin Oncol.

[CR9] Khobrekar NV, Vallee RB (2020). A RILP-regulated pathway coordinating autophagosome biogenesis with transport. Autophagy.

[CR10] Khobrekar NV, Quintremil S, Dantas TJ, Vallee RB (2020). The Dynein adaptor RILP controls neuronal autophagosome biogenesis, transport, and clearance. Dev Cell.

[CR11] Langlais P, Dong LQ, Hu D, Liu F (2000). Identification of Grb10 as a direct substrate for members of the src tyrosine kinase family. Oncogene.

[CR12] Lei Y, Xu X, Liu H, Chen L, Zhou H, Jiang J, Yang Y, Wu B (2021). HBx induces hepatocellular carcinogenesis through ARRB1-mediated autophagy to drive the G(1)/S cycle. Autophagy.

[CR13] Lin J, Zhuo Y, Yin Y, Qiu L, Li X, Lai F (2021). Methylation of RILP in lung cancer promotes tumor cell proliferation and invasion. Mol Cell Biochem.

[CR14] Liu M, Bai J, He S, Villarreal R, Hu D, Zhang C, Yang X, Liang H, Slaga TJ, Yu Y (2014). Grb10 promotes lipolysis and thermogenesis by phosphorylation-dependent feedback inhibition of mTORC1. Cell Metab.

[CR15] Liu X, Meng L, Li X, Li D, Liu Q, Chen Y, Li X, Bu W, Sun H (2020). Regulation of FN1 degradation by the p62/SQSTM1-dependent autophagy-lysosome pathway in HNSCC. Int J Oral Sci.

[CR16] Matsui T, Ohbayashi N, Fukuda M (2012). The Rab interacting lysosomal protein (RILP) homology domain functions as a novel effector domain for small GTPase Rab36: Rab36 regulates retrograde melanosome transport in melanocytes. J Biol Chem.

[CR17] Meng L, Feng B, Luan L, Fang Z, Zhao G (2022). MeCP2 inhibits ischemic neuronal injury by enhancing methylation of the FOXO3a promoter to repress the SPRY2-ZEB1 axis. Exp Mol Med.

[CR18] Mohamed AS, Hosney M, Bassiony H, Hassanein SS, Soliman AM, Fahmy SR, Gaafar K (2020). Sodium pentobarbital dosages for exsanguination affect biochemical, molecular and histological measurements in rats. Sci Rep.

[CR19] Plasschaert RN, Bartolomei MS (2015). Tissue-specific regulation and function of Grb10 during growth and neuronal commitment. Proc Natl Acad Sci USA.

[CR20] Qi C, Zou L, Wang S, Mao X, Hu Y, Shi J, Zhang Z, Wu H (2022). Vps34 inhibits hepatocellular carcinoma Invasion by regulating endosome-lysosome trafficking via Rab7-RILP and Rab11. Cancer Res Treat.

[CR21] Sapmaz A, Berlin I, Bos E, Wijdeven RH, Janssen H, Konietzny R, Akkermans JJ, Erson-Bensan AE, Koning RI, Kessler BM (2019). USP32 regulates late endosomal transport and recycling through deubiquitylation of Rab7. Nat Commun.

[CR22] Shoaib Z, Fan TM, Irudayaraj JMK (2022). Osteosarcoma mechanobiology and therapeutic targets. Br J Pharmacol.

[CR23] Steffan JJ, Williams BC, Welbourne T, Cardelli JA (2010). HGF-induced invasion by prostate tumor cells requires anterograde lysosome trafficking and activity of Na+-H+ exchangers. J Cell Sci.

[CR24] Tyagi M, Reddy D, Gupta S (2017). Genomic characterization and dynamic methylation of promoter facilitates transcriptional regulation of H2A variants, H2A.1 and H2A.2 in various pathophysiological states of hepatocyte. Int J Biochem Cell Biol.

[CR25] Vos S, Moelans CB, van Diest PJ (2017). BRCA promoter methylation in sporadic versus BRCA germline mutation-related breast cancers. Breast Cancer Res.

[CR26] Wang Z, Zhou Y, Hu X, Chen W, Lin X, Sun L, Xu X, Hong W, Wang T (2015). RILP suppresses invasion of breast cancer cells by modulating the activity of RalA through interaction with RalGDS. Cell Death Dis.

[CR27] Wang SS, Chen YH, Chen N, Wang LJ, Chen DX, Weng HL, Dooley S, Ding HG (2017). Hydrogen sulfide promotes autophagy of hepatocellular carcinoma cells through the PI3K/Akt/mTOR signaling pathway. Cell Death Dis.

[CR28] Wang X, Qin G, Liang X, Wang W, Wang Z, Liao D, Zhong L, Zhang R, Zeng Y-X, Wu Y (2020). Targeting the CK1α/CBX4 axis for metastasis in osteosarcoma. Nat Commun.

[CR29] Wang Y, Yang K, Li G, Liu R, Liu J, Li J, Tang M, Zhao M, Song J, Wen X (2020). p75NTR(−/−) mice exhibit an alveolar bone loss phenotype and inhibited PI3K/Akt/β-catenin pathway. Cell Prolif.

[CR30] Wani A, Gupta M, Ahmad M, Shah AM, Ahsan AU, Qazi PH, Malik F, Singh G, Sharma PR, Kaddoumi A (2019). Alborixin clears amyloid-β by inducing autophagy through PTEN-mediated inhibition of the AKT pathway. Autophagy.

[CR31] Whelan JS, Davis LE (2018). Osteosarcoma, chondrosarcoma, and chordoma. J Clin Oncol.

[CR32] Xie J, Ye Z, Li L, Xia Y, Yuan R, Ruan Y, Zhou X (2022). Ferrostatin-1 alleviates oxalate-induced renal tubular epithelial cell injury, fibrosis and calcium oxalate stone formation by inhibiting ferroptosis. Mol Med Rep.

[CR33] Yang M, Wei R, Zhang S, Hu S, Liang X, Yang Z, Zhang C, Zhang Y, Cai L, Xie Y (2023). NSUN2 promotes osteosarcoma progression by enhancing the stability of FABP5 mRNA via m(5)C methylation. Cell Death Dis.

[CR34] Yoshida GJ (2017). Therapeutic strategies of drug repositioning targeting autophagy to induce cancer cell death: from pathophysiology to treatment. J Hematol Oncol.

[CR35] Yu Y, Yoon SO, Poulogiannis G, Yang Q, Ma XM, Villén J, Kubica N, Hoffman GR, Cantley LC, Gygi SP (2011). Phosphoproteomic analysis identifies Grb10 as an mTORC1 substrate that negatively regulates insulin signaling. Science.

[CR36] Yu L, Wei J, Liu P (2021). Attacking the PI3K/Akt/mTOR signaling pathway for targeted therapeutic treatment in human cancer. Seminars in cancer biology.

[CR37] Zhang M, Liu S, Chua MS, Li H, Luo D, Wang S, Zhang S, Han B, Sun C (2019). SOCS5 inhibition induces autophagy to impair metastasis in hepatocellular carcinoma cells via the PI3K/Akt/mTOR pathway. Cell Death Dis.

