# Supplementary figures and images for: RILP inhibits tumor progression in osteosarcoma via Grb10-mediated inhibition of the PI3K/AKT/mTOR pathway
Source: Mol Med. 2023 Oct 3;29:133. doi: 10.1186/s10020-023-00722-6 (PMC10548720; doi:10.1186/s10020-023-00722-6)

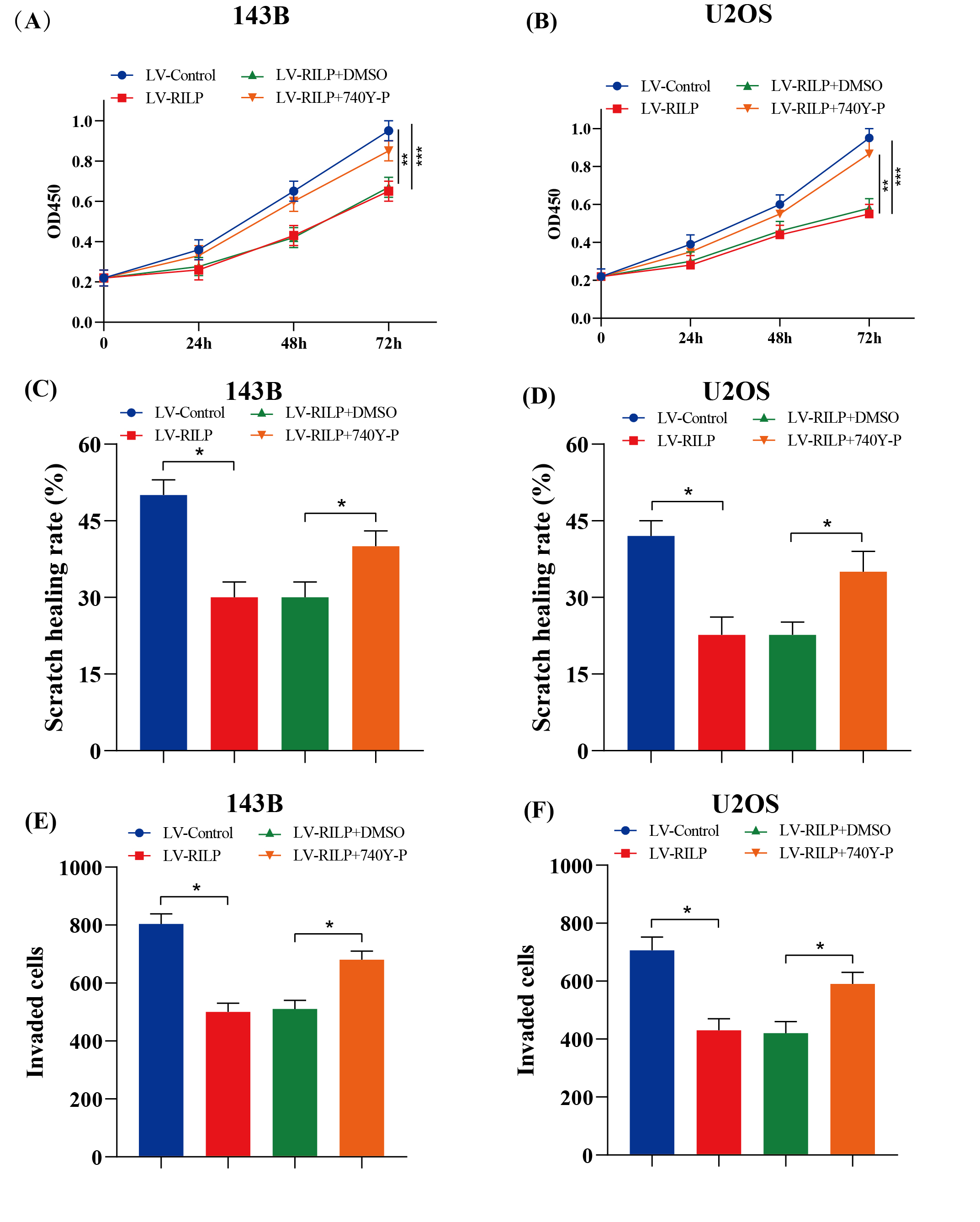

Supplement: Supplementary file 1 — Additional file 1: Figure S1. (A–F) CCK-8, wound healing, and transwell invasion assays were performed to access the effects of 740Y-P (an activator of PI3K signaling pathway) on osteosarcoma cell proliferation, migration, and invasion. *P < 0.05, **P < 0.01, ***P < 0.001. [file 10020_2023_722_MOESM1_ESM.jpg]

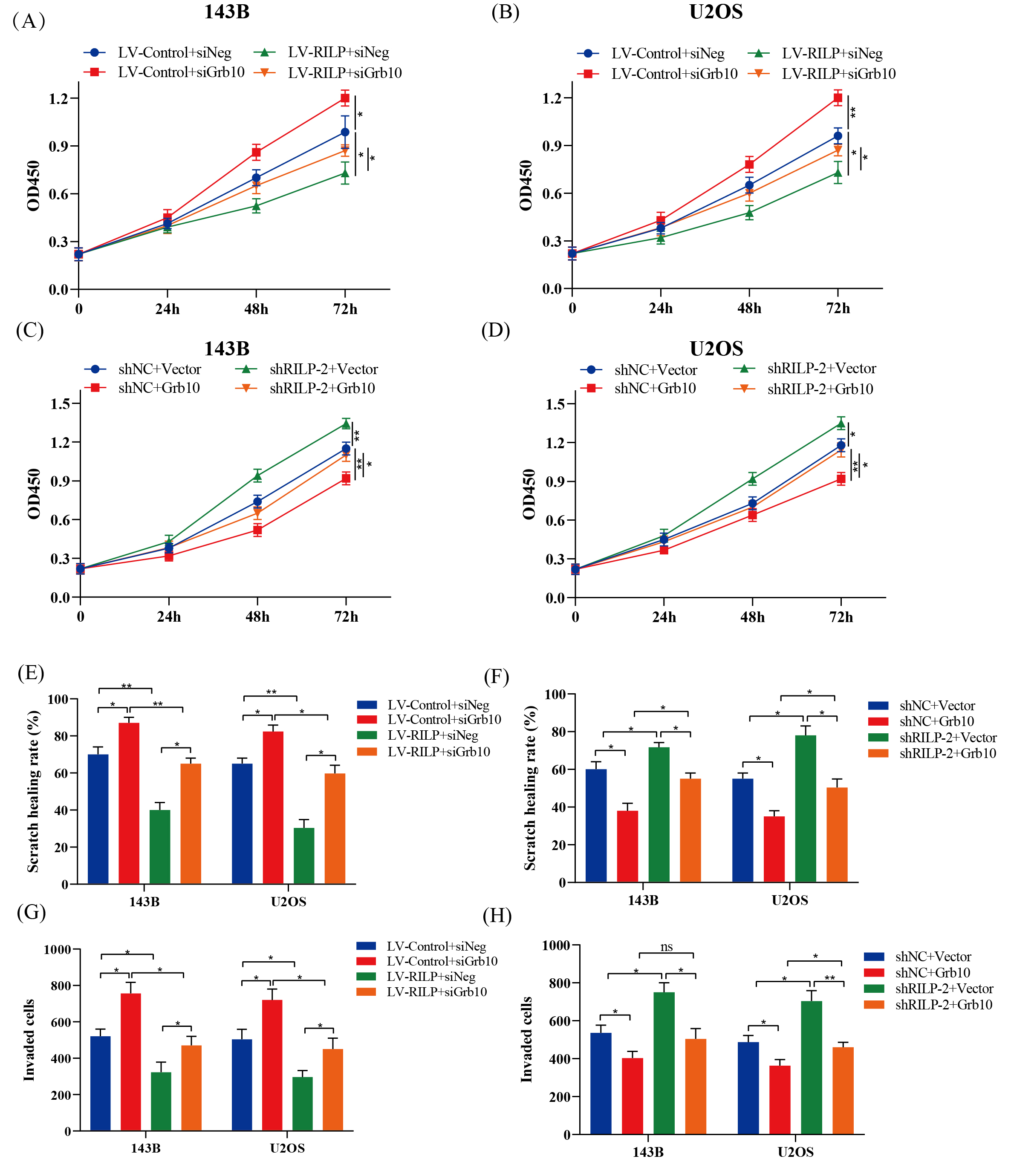

Supplement: Supplementary file 2 — Additional file 2: Figure S2. (A–H) CCK-8, wound healing, and transwell invasion assays showed that Grb10 mediated the effect of RILP on osteosarcoma cell proliferation, migration, and invasion. *P < 0.05,**P < 0.01. [file 10020_2023_722_MOESM2_ESM.jpg]
